# Retraction Note: The numerical study of pressure and temperature effects on mechanical properties of baghdadite-based nanostructure: molecular dynamics simulation

**DOI:** 10.1038/s41598-023-37408-2

**Published:** 2023-06-26

**Authors:** Qun Liu, Olga Bykanova, Ravil Akhmadeev, Shaghaiegh Baghaie, Maboud Hekmatifar, Ahmadreza Arefpour, Roozbeh Sabetvand, Vitaliy Borisov

**Affiliations:** 1 Harbin University, Harbin, 150080 Heilongjiang China; 2 Department of Higher Mathematics, Plekhanov Russian University of Economics, Stremyanny Lane, 36, Moscow, Russia 117997; 3 Department of Finance and Prices, Plekhanov Russian University of Economics, Stremyanny Lane, 36, Moscow, Russia 117997; 4 Department of Mechanical Engineering, Khomeinishahr Branch, Islamic Azad University, Khomeinishahr, Iran; 5 Advanced Materials Research Center, Department of Materials Engineering, Najafabad Branch, Islamic Azad University, Najafabad, Iran; 6 Department of Energy Engineering and Physics, Faculty of Condensed Matter Physics, Amirkabir University of Technology, Tehran, Iran; 7 Department of Propaedeutics of Dental Diseases, Sechenov First Moscow State Medical University, Moscow, Russia

Retraction of: *Scientific Reports* 10.1038/s41598-022-11642-6, published online 07 May 2022

Editors have retracted this Article.

After publication, concerns were raised about authorship and description of author contributions, as well as nonsensical phrases used in the Article and an overlap with a previously-published paper that shares 3 authors in common^1^. The Editors requested the Authors to provide raw original data and explanations regarding the contributions and the overlap but found the response provided by the Authors insufficient. Given these issues and that authorship of the paper could not be verified, the Editors no longer have confidence in the content of this Article.

Shaghaiegh Baghaie disagrees with this retraction. Qun Liu did not respond to the correspondence about this retraction. The Editors have not been able to establish current email addresses for Olga Bykanova, Ravil Akhmadeev, Maboud Hekmatifar, Ahmadreza Arefpour, Roozbeh Sabetvand & Vitaliy Borisov.
